# Retraction Note: Perioperative left ventricular perforation in incomplete TAVI and completion of the procedure after surgical repair

**DOI:** 10.1186/s13019-024-03045-7

**Published:** 2024-09-20

**Authors:** Giuseppe Nasso, Giuseppe Santarpino, Gaetano Contegiacomo, Giuseppe Balducci, Antongiulio Valenzano, Enrico Moranti, Domenico Scaringi, Giuseppe Speziale, Ignazio Condello

**Affiliations:** 1 Department of Cardiac Surgery, Anthea Hospital, GVM Care and Research, Via Camillo Rosalba 35/37, 70124, Bari, Italy; 2 Department of Cardiac Surgery, Paracelsus Medical University, Nuremberg, Germany; 3 Cardiac Surgery Unit, Department of Experimental and Clinical Medicine, “Magna Graecia” University of Catanzaro, Catanzaro, Italy; 4 Department of Interventional Cardiology, Anthea Hospital, GVM Care and Research, Bari, Italy

**Retraction Note: Journal of Cardiothoracic Surgery (2022) 17:171**

10.1186/s13019-022-01925-4

The Editor-in-Chief has retracted this article because it overlaps significantly in text and case details with a previously published article with no common authors [1]. The Editor-in-Chief no longer has confidence in the reliability of the reporting of this case.

Giuseppe Nasso and Ignazio Condello disagree with this retraction. The remaining authors did not respond to correspondence from the Publisher about this retraction.
